# Identification of two new GRAS transcription factors and expression analysis of these genes in *Chenopodium quinoa*

**DOI:** 10.3389/fpls.2025.1579338

**Published:** 2025-07-18

**Authors:** Xinwen Hou, Shuwei Wang, Shanmin Zhou, Weizhong Liu

**Affiliations:** ^1^ School of Chemistry and Material Science, Shanxi Normal University, Taiyuan, China; ^2^ School of Life Science, Shanxi Normal University, Taiyuan, China; ^3^ College of Life Sciences and Oceanography, Shenzhen University, Shenzhen, China

**Keywords:** GRAS genes, abiotic stresses, *Chenopodium quinoa*, gene expression, genome-wide identification

## Abstract

*Chenopodium quinoa* is a relatively new and excellent crop, and its growth is frequently threatened by abiotic stress. *GRAS* genes are considered to be a plant-specific transcriptional regulatory family, which is essential for controlling aboveground and root development, as well as enhancing tolerance to abiotic stress. Phylogeny, gene structure, genomic location, conserved motif, cis-element, protein interaction, and expression pattern were all comprehensively investigated in this research of the quinoa *GRAS* genes. According to its structure and phylogenetic characteristics, the identified quinoa 54 GRAS members were divided into 10 subgroups. The distribution of *CqGRAS* genes on 19 quinoa chromosomes is uneven, with Chr07 and Chr18 having the largest number of genes. The quinoa GRAS family’s evolution has been driven by duplication and collinearity among members. Under abiotic stress, 12 selected *CqGRAS* genes showed significant differential expression. *CqGRAS1* and *19* were most sensitive to low temperatures, H_2_O_2_ treatment highly induced the expression of *CqGASS20*, and Na_2_CO_3_ treatment highly induced the expression of *CqGRAS23*. After conducting tissue quantification, we found that some *CqGRAS* genes exhibit tissue-specific expression patterns, with *CqGRAS19* and *45* being highly expressed in stems and *CqGRAS3* and *32* being highly expressed in leaves. In summary, this work gives valuable information for a comprehensive understanding of the functional analysis of the *Chenopodium quinoa* genome’s *GRAS* gene family and the identification of candidate genes to improve quinoa’s resistance to abiotic stress.

## 1 Introduction

The medium- and high-altitude mountains of the Andes in South America are the original home of *Chenopodium quinoa* (Bazile et al., 2021). It has been cultivated and eaten for more than 6,000 years, almost at the same time as rice, and it has good stress resistance after years of evolution. This is closely related to the fact that it grows in plateaus or mountain areas at an altitude of 3,000-4,000 meters above sea level. *Chenopodium quinoa* has comprehensive nutrition, a unique taste, and strong resilience for extreme weather as well as soils, especially drought, saline-alkali, and cold (Jacobsen et al., 2007; Yang et al., 2016; Dehghanian et al., 2024). It is an ideal candidate material for desert, saline-alkali soil growth, and stress resistance (Hinojosa et al., 2018). The release of the genome sequence provides a basis for accelerating genetic improvement and screening of *Chenopodium quinoa* crops (Jarvis et al., 2017; Zou et al., 2017).

By binding themselves to downstream DNA, transcription factors can influence the level of expression of genes, thus adapting to external adverse conditions. GAI (gibberellin insensitive), RGA (GA1–3 mutant repressor), and SCR (scarecrow) are the first three members of this family, and GRAS proteins are plant transcription factors that bear their names (Bolle, 2004). Typically, they have only one GRAS domain, but a few have two. Its C-terminal and N-terminal contain 5 and 2 highly conserved motifs with different functions, respectively. PFYRE, SAW, VHIID, leucine-rich region I (LHRI), as well as leucine-rich region II (LHRII), make up the C-terminal. A crucial function of LHRI and LHRII is to homodimerize the GRAS protein (Niu et al., 2019). The core component of the GRAS protein is the VHIID motif (Hofmann, 2016). Extremely conservative, the P-N-H-D-Q-L in the motif concludes in L-R-I-T-G. The P, FY, and RE that make up the PFYRE motif might have something to do with phosphorylation. The three pairs of conserved amino acid residues that were discovered in the SAW motif may be related to the GRAS protein’s integrity. The N-terminal of the GRAS protein has two highly conserved protein structures, DELLA and TVHYNP, which are highly variable regions. In order to bind to the target protein and take part in the signal transduction process, the amino acid sequence could be folded into a special molecular recognition structure (Sun et al., 2012). According to the structural characteristics, GRAS proteins are divided into 10 subfamilies: DELLA, AtLAS, AtSCR, AtSHR, AtPAT1, HAM, LISCL, AtSCL3, SCL4/7, and DLT (Sun et al., 2011). The number of GRAS subfamilies varies in different species. It can be divided into 17 branches in bottle gourd (Sidhu et al., 2020) and birch (He et al., 2022), 16 branches in garlic (Zhang et al., 2022), 14 branches in cassava (Shan et al., 2020) and wheat (Mishra et al., 2023), 12 branches in barley (To et al., 2020) and cacao (Hou et al., 2022), and 13 branches in poplar (Liu and Widmer, 2014), sorghum (Fan et al., 2021b), foxtail millet (Fan et al., 2021a), rye (Fan et al., 2024), and eggplant (Yang et al., 2022), more than previously proposed branches.

GRAS transcription factors regulate many development processes, such as light signal transduction, meristem maintenance, root radial pattern formation, and stress response (Yuan et al., 2016; Feng et al., 2021; Waseem et al., 2022). *GAI*, *RGA*, and *RGL* genes were found to inhibit gibberellin signals in the *Arabidopsis thaliana* DELLA subfamily (Wen and Chang, 2002). *SCR* and *SHR* have been reported to form the SCR/SHR complex, and for plant cortical cell division, the SHR-SCR molecule is both a necessary and sufficient condition. When SHR-SCR molecular is overexpressed in the legume alfalfa root, cortical cell division can be induced to form a root nodule-like structure. Ectopic overexpression of SHR-SCR molecular modules in non-legume *Arabidopsis* and rice roots can also induce root and cortical cell division. It is suggested that the acquisition of the SHR-SCR stem cell program module by cortical cells of leguminous plants may be a required event for symbiotic nodulation and nitrogen fixing of leguminous plants (Dong et al., 2021). *PAT1* and *SCL21* can positively regulate the signal transduction of phytochrome A (Torres-Galea et al., 2013), and *SCL13* functions independently in the phytochrome B pathway (Torres-Galea et al., 2006). Furthermore, the *SCL6* triplet mutant displayed a polymorphic phenotype similar to that of meristematic tissue cells when *miR171* was overexpressed (Wang et al., 2010). Moreover, there are a variety of abiotic stress responses involving GRAS transcription factors. In rice, maize, and *Arabidopsis thaliana*, for instance, the genes *HcSCL13*, *ZmSCL7*, *AtRGA*, and *AtGAI* have been responding to salt stress (Zeng et al., 2019; Zhang et al., 2020). *VaPAT1* regulates JA biosynthesis and serves a significant part in the grapevine’s response to cold stress, and *Arabidopsis* seedlings have increased levels of proline and soluble sugars while overexpressing, improving resistance to cold, dehydration, and salt (Yuan et al., 2016; Wang et al., 2021), and *NtGRAS1* in tobacco can increase the level of ROS under various stresses (Czikkel and Maxwell, 2007). More importantly, the *Arabidopsis thaliana* protein SCL14 is necessary for stress-related promoter activation (Fode et al., 2008). In addition, high expression of *GmGRAS37* in soybean can promote the resistance of soybean hairy roots to drought and salinity treatment, and *OsGRAS23* can induce numerous genes to positively regulate the drought tolerance of rice (Xu et al., 2015). The poplar *GRAS/SCL* gene *PeSCL7* had a similar stress response to grape *VaPAT1*, which was blocked by gibberellin and triggered by excessive salt and dryness. When *PeSCL7* was overexpressed, *Arabidopsis thaliana* exhibited higher resistance to drought and salt (Ma et al., 2010).

GRAS family proteins have just recently been identified in a small number of plants, such as *Arabidopsis thaliana* (Tian et al., 2004), rice (Dutta et al., 2021), tomato (Huang et al., 2015), potato (Chen et al., 2019), *Populus* (Liu and Widmer, 2014), lotus (Wang et al., 2016), grape (Grimplet et al., 2016), *Prunus mume* (Lu et al., 2015), alfalfa (Zhang et al., 2021), castor bean (Xu et al., 2016), oat (Pan et al., 2023), Chinese chestnut (Yu et al., 2022), ginger (Tian et al., 2022), pigeonpea (Rana et al., 2023), sugar beet (Hao et al., 2024), and pine (Abarca et al., 2014). Zhu et al. identified 52 GRAS transcription factors in quinoa (Zhu et al., 2021b). Given quinoa’s remarkable stress tolerance in its unique growth environment, it is of great significance to investigate the potential resistance genes within the quinoa *GRAS* family. Our study focuses more sharply on gene expression analysis. In this study, we identified the possible *GRAS* genes in quinoa and conducted a detailed study of their phylogeny, genome structure, duplication repetition, promoter, and expression analysis. Our findings not only provide a more comprehensive and in-depth analysis of the *GRAS* gene family in quinoa but also offer a clearer direction for subsequent functional validation and genetic improvement.

## 2 Materials and methods

### 2.1 Identification of GRAS family members in *Chenopodium quinoa* and other species

The genome annotation files for *Arabidopsis*, rice, and *Chenopodium quinoa* were obtained from the Phytozome V13 Database (https://phytozome-next.jgi.doe.gov/). The profile of the hidden Markov model (HMM) (PF03514.13) was downloaded. Then, using the HMM profile, the program HMMER 3.1 conducted HMM searches against annotated protein databases from various genomes with an E-value cutoff of 1e−5 (Finn et al., 2011). The Conserved Domain Database at the National Center for Biotechnology Information (NCBI) (https://www.ncbi.nlm.nih.gov/) was then used to confirm the conserved GRAS domain of all potential *Chenopodium quinoa* GRAS. Furthermore, we arranged GRAS proteins’ pIs, lengths, weights, chrs, and related gene positions. The PROT-PARAM tool (http://web.expasy.org/protparam/) was used to estimate the physical and chemical characteristics of proteins, including molecular weight and grand average of hydropathy (GRAVY). The Wolf PSORT online tool (https://wolfpsort.hgc.jp/) was used to predict the subcellular location of proteins.

### 2.2 Phylogenetic analysis of GRAS members

The MAFFT online program (https://mafft.cbrc.jp/alignment/server/) was used to align all of the protein sequences in this study. The full GRAS protein sequences in *Arabidopsis thaliana* and *Chenopodium quinoa* were utilized for this alignment. The FFT-NS-I algorithm was used, and the parameter of the scoring matrix for amino acid sequences was set to BLOSUM62 (Katoh and Standley, 2013). The neighbor-joining (NJ) method was then used to perform phylogenetic analysis on the aligned sequences. Using MEGA X and the pairwise deletion option with 1000 bootstrap replicates, the consensus tree was constructed using the JTT model (Kumar et al., 2018). Finally, we split the subfamily based on earlier results and used ITOL software (https://itol.embl.de/) to modify and visualize the phylogenetic trees (Letunic and Bork, 2019).

### 2.3 Chromosomal localization and gene duplication

We thoroughly investigated the Phytozome V13 database for the chromosomal locations of the *Chenopodium quinoa GRAS* genes. In accordance with earlier reports, duplicate gene pairs were carefully examined and illustrated using the TBtools software (Chen et al., 2020; To et al., 2020). DnaSP v6.0 software was used to estimate the Ka (non-synonymous substitution rate) and Ks (synonymous substitution rate) (Rozas et al., 2017). The Ka/Ks ratio was calculated to determine the selection pressure for each pair of duplicated genes.

### 2.4 Analysis of the conserved motifs and gene structures

By supplying a relevant map that was taken from the genomic GFF3 map, TBtools is used to visualize the gene structure (intron and exon) of the GRAS genes. The conserved motifs on the protein sequences were then predicted using the MEME website (http://meme-suite.org/). A total of ten motifs were identified, and other parameters were set to the website’s default values (Bailey et al., 2015). The motifs on protein sequences were visualized using the TBtools software (Chen et al., 2020). Through the study of *microRNA171*, the target site of miRNA in the quinoa GRAS family was obtained.

### 2.5 Analysis of cis-acting elements in the promoter regions

The upstream 2,000 bp promoter sequence of each *CqGRAS* gene was first extracted from the *Chenopodium quinoa* genome. Then, the sequence was submitted to the online tool PlantCare (http://bioinformatics.psb.ugent.be/webtools/plantcare/html/) to predict the cis-acting elements in the *CqGRAS* promoter region (Lescot et al., 2002). The number of cis-acting elements was calculated using Excel.

### 2.6 PPI network of GRAS protein in *C. quinoa*

To further illustrate the interactions between CqGRAS proteins, the network of interactions between proteins was predicted using orthologues of *Arabidopsis*. A network of functional interactions between proteins was created using the STRING website (https://www.string-db.org/), with a confidence parameter set to 0.15. The network was visualized using Cytoscape 3.7.2 (Su et al., 2014).

### 2.7 Plant materials and stress treatments

YmsBLM-2 white *Chenopodium quinoa* seeds were generously provided by the College of Life Sciences, Shanxi Normal University. A growth chamber with restricted circumstances (25°C day/22°C night, 16 hours light/8 hours dark) was used for cultivating sterilized seeds. One-month-old *Chenopodium quinoa* plants were exposed to several abiotic stress treatments. The cold treatment involved putting 4-week-old quinoa in a light incubator set at 4°C for 0, 2, 4, 6, 8, 10, and 12 hours, respectively. Quinoa was transplanted to 1/2 MS medium with varying H_2_O_2_ and Na_2_CO_3_ concentrations (0, 5, 10, 15, and 20% for H_2_O_2_; 0, 2, 4, 6, and 8 mM for Na_2_CO_3_) for 6 hours. Each sample included three separate biological replications.

### 2.8 Plant materials and quantitative real-time PCR

A growth chamber with restricted circumstances (25°C day/22°C night, 16 hours light/8 hours dark) was used for cultivating sterilized seeds. RNA samples were isolated from the roots, stems, and leaves of seedlings when they were four to five leaves old. The RNeasy Plant Mini Kit (TransGen Biotech, Beijing, China) was then used to extract the total RNAs, and the SuperScript™ III Reverse Transcriptase kit (Invitrogen) was used to prepare the cDNA. The primers used were designed using Primer 5.0 (**Supplementary Table S1**). Quantitative real-time PCR (qRT-PCR) was carried out using the 2× Perfect SYBR Green PCR Mix (TransGen Biotech, Beijing, China) and the QuantStudio 3 PCR System (Life Technologies, Singapore), strictly adhering to the manufacturer’s instructions. The qRT-PCR instrument was set to 40 cycles with a 60°C annealing temperature. Relative gene transcript levels were measured as 2^−⊿⊿Ct^ and normalized against those of *Elongation Factor 1 alpha* (*EF1α*) (Livak and Schmittgen, 2001; Vandesompele et al., 2002; Zhu et al., 2021a).

### 2.9 Statistical analyses

Each experiment was repeated at least three times. Statistical analysis was performed with analysis of variance (ANOVA) using GraphPad Prism 8 (https://www.graphpad.com/scientific-software/prism/). Test differences were determined to be significant with a *P*-value cut-off of 0.05. Results were expressed as mean ± s.d.

## 3 Results

### 3.1 Identification of *CqGRAS* genes in *Chenopodium quinoa*

Drawing from the GRAS family’s conserved data, we identified 54 *CqGRAS* genes in the *Chenopodium quinoa* genome using a variety of methods. Then rename them *CqGRAS1* to *CqGRAS54* (**Supplementary Table S2**). Some information about the family proteins is listed as follows: isoelectric point (pI), hydrophilicity and subcellular localization, protein molecular weight (MW), and other basic characteristics (**Supplementary Table S3**). Among the 54 CqGRAS proteins, the largest, CqGRAS33, has 1,487 amino acids, while the smallest, CqGRAS41, has 229 amino acids. Proteins with molecular weights between 25.6 (CqGRAS41) and 168.3 (CqGRAS33) kDa, and the isoelectric point is 4.65 (CqGRAS19) ~ 9.2 (CqGRAS51), with an average of 5.97. The hydrophilic values were all less than zero except for CqGRAS41, which was 0.067, indicating that CqGRAS41 was a hydrophobic protein while all the others were hydrophilic proteins. The range of the *CqGRAS* genes’ CDS lengths is 690 bp to 4,302 bp. At 690 bp, the CDS of *CqGRAS41* is the shortest, and the CDS of *CqGRAS33* is the longest. The predicted subcellular localization results suggested that 20 CqGRAS proteins were located in the nuclear region, 16 in the cytoplasm, 17 in the chloroplast, and 1 in the plasma membrane. In our study, the CqGRAS5 protein was predicted to be localized to the plasma membrane, which may indicate that it has functions different from typical transcription factors, such as participating in signal transduction or intercellular communication.

### 3.2 Phylogenetic analysis and classification of *CqGRAS* genes

**Figure 1:**
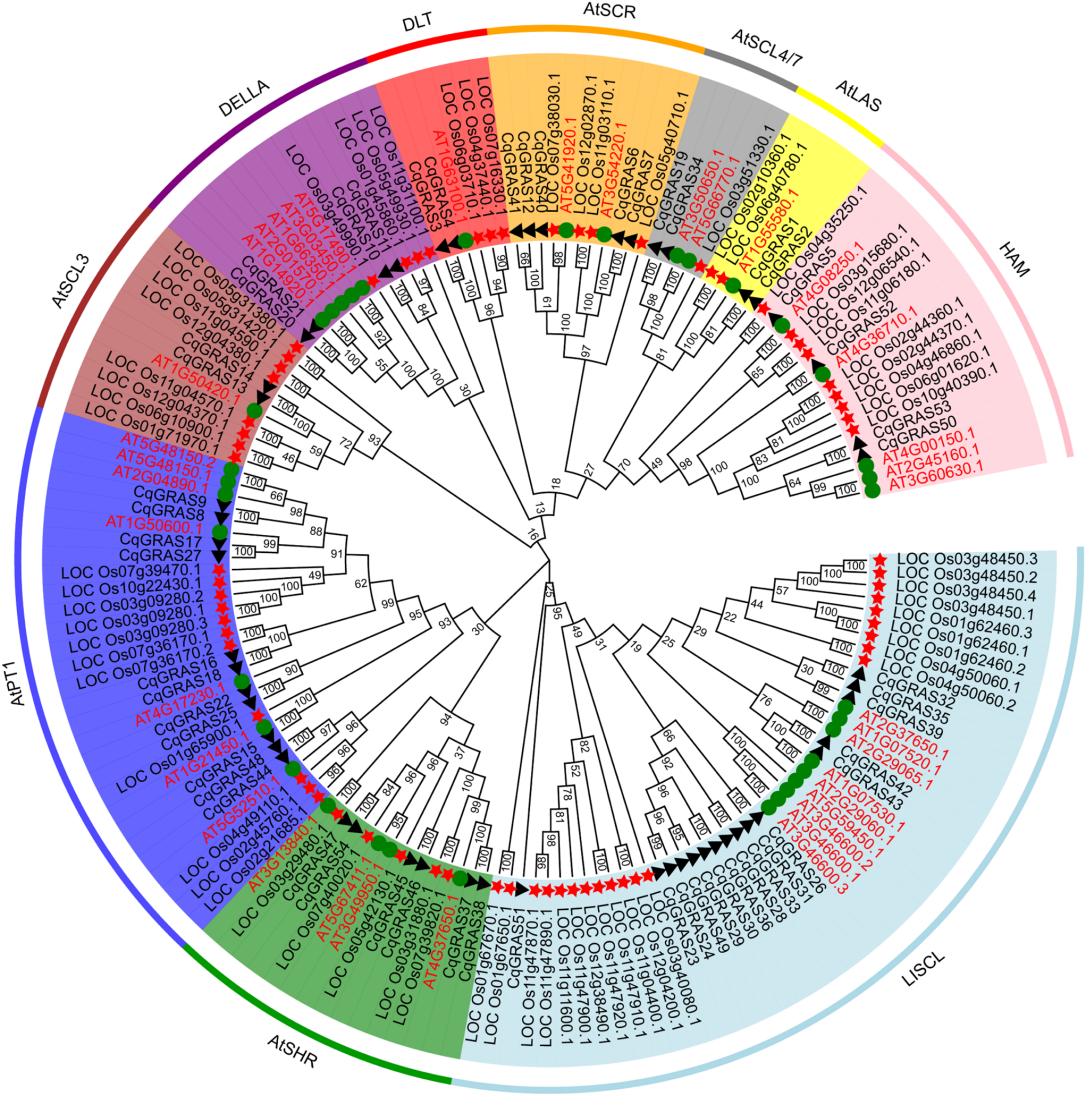
*CqGRAS* gene phylogenetic tree in rice, *Arabidopsis*, and *Chenopodium quinoa*. The neighbor-joining method was used to create the phylogenetic tree, which has 1000 bootstrap repetitions. Different colors are used to indicate 10 classifications, and each subfamily’s name is also indicated in the corresponding place. Different shapes are used for the representation of the *GRAS* genes of the three species: triangle for *Chenopodium quinoa*, star for rice, and circle for *Arabidopsis*.

To investigate the phylogenetic relationship and classification of *GRAS* genes in *Chenopodium quinoa*, 54 protein sequence alignments served as the basis for the phylogenetic tree we created, including 69 rice protein sequences and 37 *Arabidopsis* protein sequences (**Figure 1**; **Supplementary Table S4**). The neighbor-joining method was used to create the phylogenetic tree. Based on the phylogenetic relationship of GRAS proteins in *Arabidopsis thaliana*, the CqGRAS proteins were classified into 10 subfamilies: HAM, SHR, SCL3, LAS, SCR, DLT, SCL4/7, DELLA, PAT1, and LISCL. There are 16 *CqGRAS* genes in the LISCL subfamily, which is the largest. DLT, LAS, SCL3, and SCL4/7 subfamilies all have just 2 members. PAT1, HAM, DELLA, SCR, and SHR have 11, 4, 4, 5, and 6 *CqGRAS* genes, respectively. Similar to other species, *Chenopodium quinoa* has a higher number of GRAS transcription factors from the PAT1, SHR, HAM, and DELLA subfamilies than from other subfamilies. The DELLA subfamily contains 5 *Arabidopsis thaliana*, 4 *Chenopodium quinoa*, and 4 rice *GRAS* genes. *Chenopodium quinoa GRAS* genes are distributed similarly to those of *Arabidopsis thaliana* in other subfamilies, and the orthologous genes can be well clustered together, indicating that the evolutionary relationship between *Chenopodium quinoa GRAS* genes and *Arabidopsis thaliana* is closer than that of rice, so the reference value of *Arabidopsis GRAS* genes is higher. We can infer the function of these unknown genes since members of the same subfamily share comparable structures and activities. In *Arabidopsis thaliana*, for instance, *AT3G03450.1* and *AT1G66350.1*, members of the same subfamily, are crucial in cold stress. Therefore, we speculate that quinoa DELLA subfamily genes (such as *CqGRAS20*) contribute to the control of self-protection in low-temperature environments.

### 3.3 Chromosomal localization and gene duplication analysis of *CqGRAS* genes

**Figure 2:**
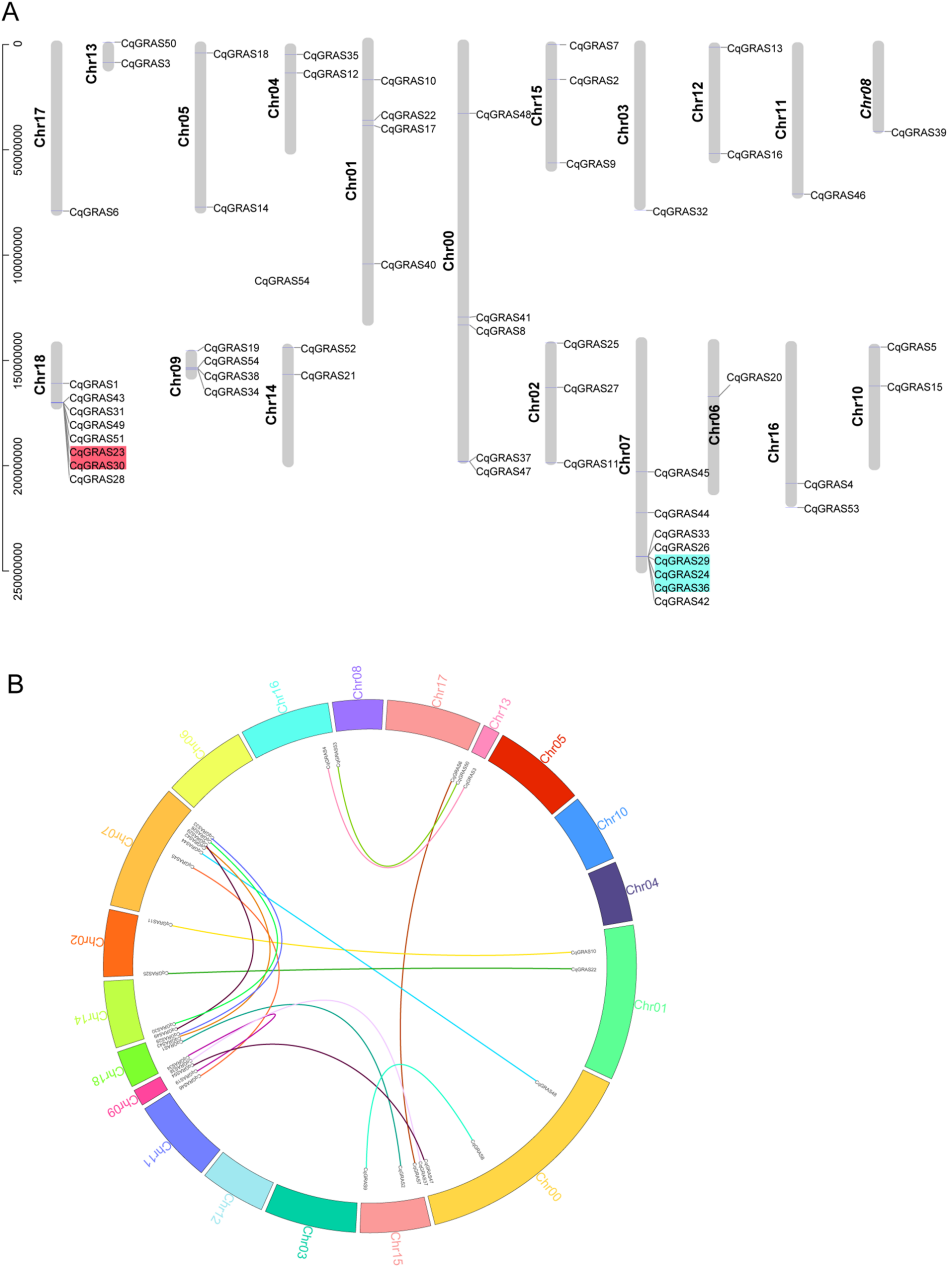
Chromosomal localization and gene duplication analysis of *CqGRAS* genes. **(A)** Positions of *GRAS* gene family members on quinoa chromosomes. A different-colored background indicates tandem duplicated genes. The physical positions of *CqGRAS* genes determine their locations. **(B)** Inter-chromosomal relations of the *CqGRAS* genes. Chromosome number is shown by the colored text. The colorful lines linked the duplicated *CqGRAS* gene pairs.

Based on the quinoa genome’s location data, the chromosome mapping map of the *CqGRAS* gene family was constructed (**Figure 2A**). The findings revealed that the 19 quinoa chromosomes have an unequal distribution of *CqGRAS* genes. The *CqGRAS* genes were most abundant on Chr07 and Chr18 (with 8 genes on each chromosome, accounting for 26%), and they exhibited a clustered distribution. Secondly, there are different numbers of genes on other chromosomes. Nevertheless, Chr03, 06, 08, 11, and 17 contained only one *GRAS* gene. It is noteworthy that a large number of *CqGRAS* genes are found at both chromosomal ends, which is consistent with that in other species (Liu et al., 2018; Sidhu et al., 2020; To et al., 2020; Zhang et al., 2021). Furthermore, we investigated the *CqGRAS* genes’ duplication events since gene duplication is essential for the occurrence of new functions and the amplification of gene families. Tandem duplication events are described as chromosomal areas that are 200 kb in size and contain two or more genes. On chromosomes 7 and 18, five *CqGRAS* genes were clustered into two tandem repeat regions, demonstrating that they are hot spots in the distribution of *CqGRAS* genes. Meanwhile, 9 chromosomes were found to have 16 pairs of fragment repetitive events (**Figure 2B**). The events of tandem duplication and segmental duplication of *CqGRAS* genes mainly occurred in SCR and LISCL. We also calculated the Ka to Ks ratio of homologous fragment gene pairs and found that it was below one, indicating that purifying selection was undertaken in quinoa’s *GRAS* gene (**Supplementary Table S5**).

### 3.4 Analysis of gene structure and conserved motif

**Figure 3:**
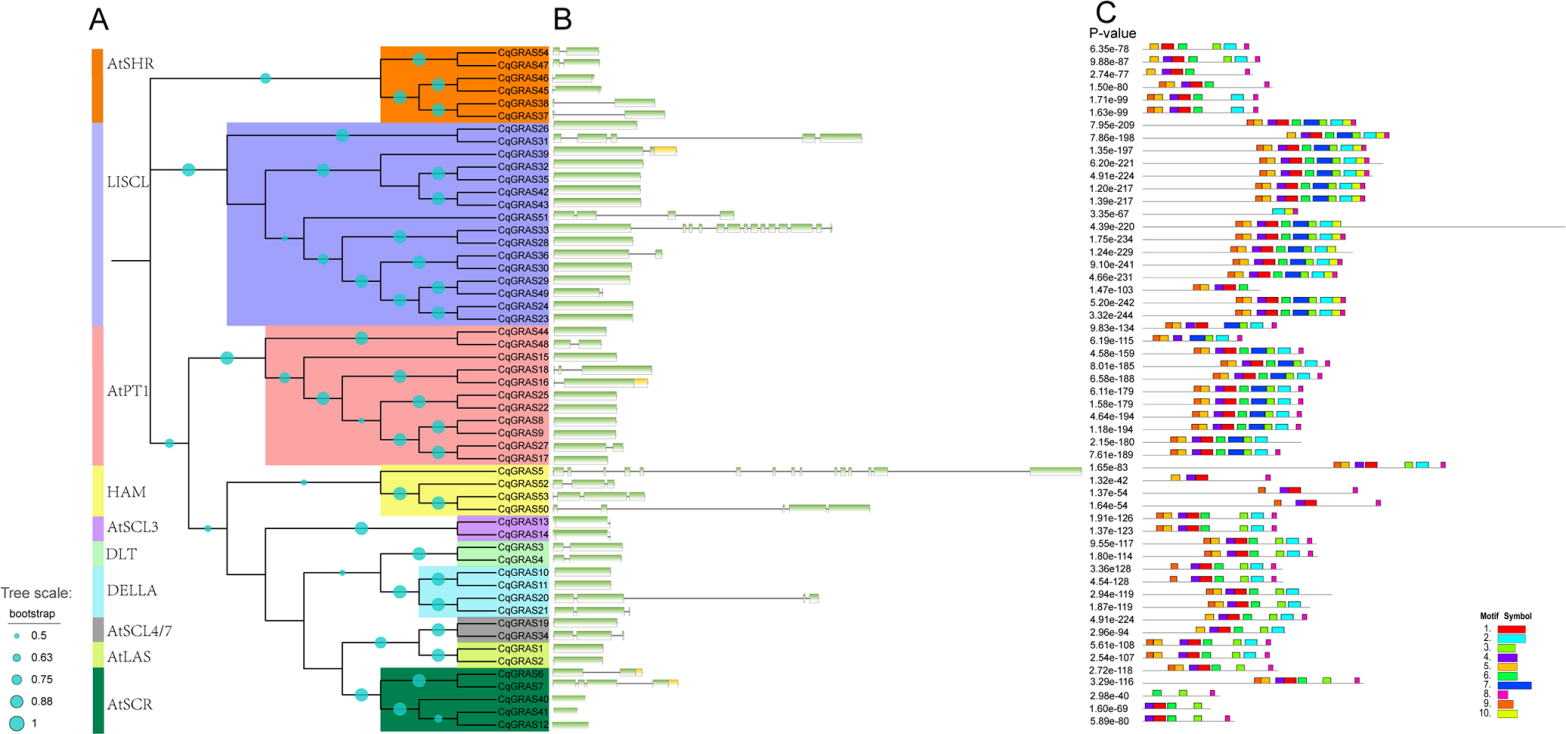
Distribution of conserved motifs within GRAS proteins in *Chenopodium quinoa*. **(A)** The phylogenetic tree was constructed by MEGA X with the neighbor-joining method. Different subfamilies are marked in different colors. **(B)** Structures of the 54 putative *Chenopodium quinoa GRAS* genes. The exons and introns are represented by boxes and black lines, respectively. **(C)** Motif distribution of GRAS proteins. The different motifs are indicated by different colors, and the combined *P*-values are shown on the left side of the figure. The same color within different proteins refers to the same motif.

Comparing the related genomic DNA sequences allowed for the determination of the exon and intron structure of the *CqGRAS* genes (**Figures 3A, B**). 54 *CqGRAS* genes contain the GRAS domain; about half of the *CqGRAS* genes (41%) have no introns, and *CqGRAS32*, *6*, *16*, *3*, *4*, *48*, *27*, and *49* contain one intron; *CqGRAS18*, *21*, and *34* have two introns. Mainly, genetic structures are similar between members of the same subfamily. The C-terminal GRAS domain of each subfamily of GRAS proteins is largely conserved, while the N-terminal is varied, which has been found in other plants, such as bottle gourd, *Arabidopsis*, poplar, and tomato (Sidhu et al., 2020). Similar component motifs can be found in the same subfamily. For instance, the members of AtSHR share the same motif, indicating a close evolutionary relationship among CqGRAS members within the same subfamily. Match these motifs with the corresponding GRAS domains. There are five distinct motifs found in the C-terminal domain of the CqGRAS protein: LR I, SAW, LR II, PFYRE, and VHIID (**Figure 3C**), but not all protein sequences contain these five sequences. Among them, LR I (motif 9) as well as LR II (motifs 1 and 3) are two leucine-rich regions, which exist in most subfamilies. At the same time, there is a VHIID domain (motifs 5 and 4) between LR I and LR II in all CqGRAS proteins, such as the PAT1 family. For another, the N-terminal DELLA domain is present in every member of the DELLA subfamily. The RE (Motif 7) unit was absent from all members of the DLT subfamily, six members of the AtSHR subfamily, and four members of the HAM subfamily. *CqGRAS40* (AtSCR subfamily) contains only motif 3, motif 6, and motif 8, which is the least number of the motifs. Motif 10 only exists in the members of the LISCL subfamily. In the sequence, the two subfamilies of LISCL and PAT1 contain 10 motifs, the most, and the others are less than 10. What is interesting is that there are significant differences in motif among different subfamilies, but there is little change within the subfamilies.

### 3.5 Analysis of cis-acting elements in the promoters of *CqGRAS* genes

**Figure 4:**
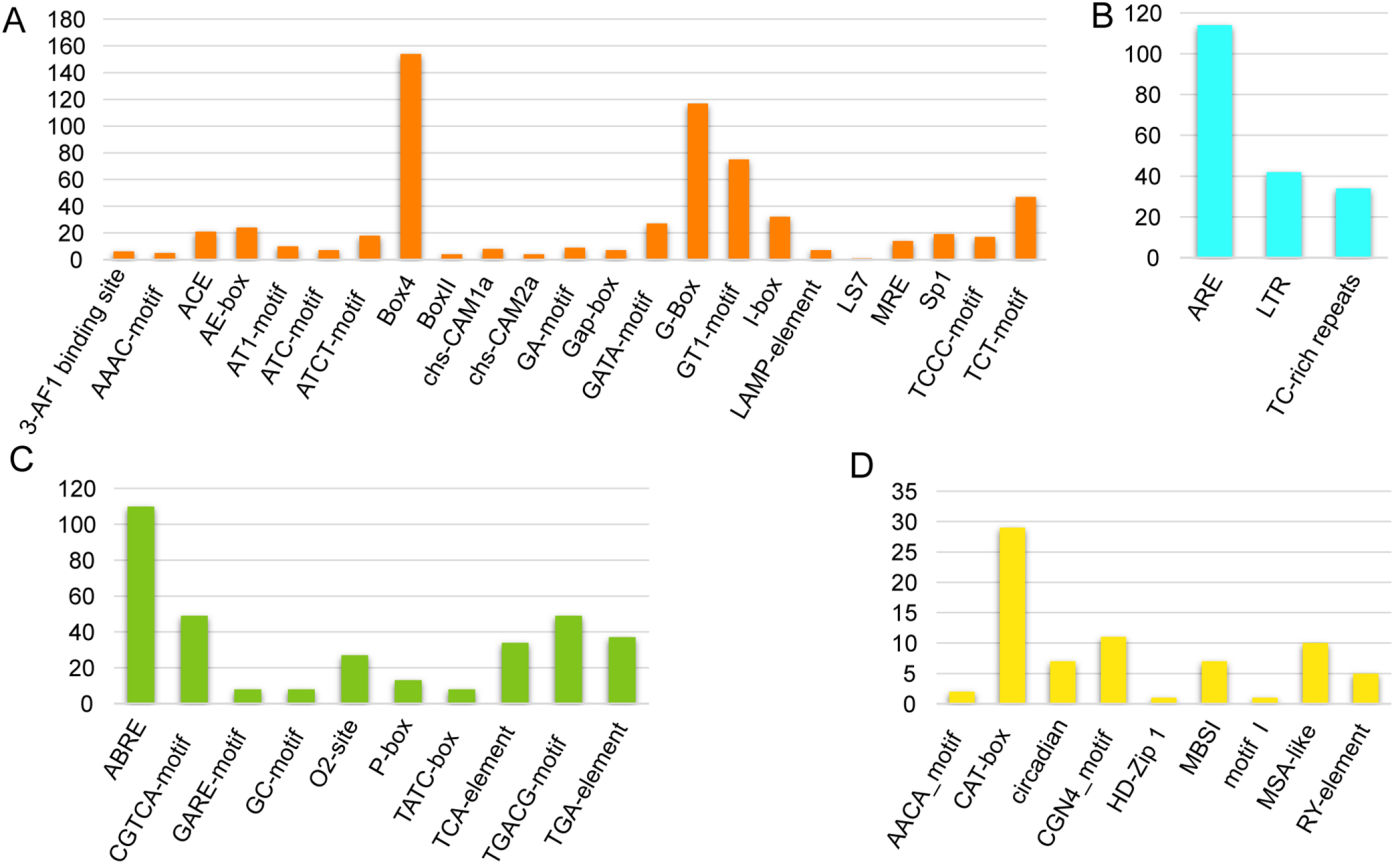
Cis-elements predicted in *CqGRAS* promoters. PlantCARE analyzed 54 *CqGRAS* genes’ promoter sequences (−2 kb). **(A)** Cis-acting elements involved in abiotic stress. **(B)** Cis-acting elements involved in light response. **(C)** Cis-acting elements involved in plant hormone response **(D)** Cis-acting elements involved in plant development and growth. The Y-axis indicates the number of each of the cis-acting elements; the X-axis indicates the different cis-acting elements.

The existence of distinct cis-acting elements inside the gene promoter may indicate that these genes have distinct roles. In order to investigate the cis-acting elements in the *CqGRAS* gene’s promoter, the genomic sequence of 2.0 kb upstream of the transcriptional initiation site of each gene was obtained and analyzed in the PlantCare database. **Figure 4** illustrates the identification and counting of cis-acting elements in all *CqGRAS* gene promoters in quinoa that are involved in plant development and growth, plant hormone response, light response, and abiotic stress. There are 9 cis-acting elements that are important in plant growth and development; the majority of quinoa *CqGRAS* genes (84.6%) have CGN4_motif elements, which participate in endosperm gene expression. In addition, the circadian rhythm element ACE is present in the promoters of most *CqGRAS* genes, such as *CqGRAS8* and *CqGRAS40*. In this study, the presence of ACE elements suggests that these genes may be regulated by the circadian clock. This regulation is crucial for coordinating the functions of these genes with the daily cycle, and may affect the growth, development, and stress response of quinoa. Studies have shown that circadian regulation of gene expression can enhance stress tolerance in other plants by optimizing the activation time of stress-response genes (Venkat and Muneer, 2022; Ahn et al., 2025). The cis-regulatory element ABRE, which was discovered in 66.7% of *CqGRAS*, is associated with *PP2C* gene promoter binding and abscisic acid induction. There are three kinds of stress response elements: ARE, LTR, and TC-rich repeats. ARE is the most widely distributed, and it is associated with anaerobic induction, whereas LTR is engaged in responses to low-temperature stress. A TC-rich repeat element has been shown to significantly increase the transgenic *Arabidopsis* plants’ tolerance to salt and drought stress, decrease the level of hydrogen peroxide (H_2_O_2_) and superoxide anion radical (O^2-^), and increase the activity of the antioxidant enzyme system. ROS-related and stress-responsive genes’ expression was up-regulated during osmotic stress to improve their ability to scavenge ROS (Zhang et al., 2018), while there were a large number of TC-rich repeats upstream of the quinoa *CqGRAS* genes, which revealed that quinoa-related genes may have similar resistance functions.

### 3.6 PPI network of GRAS protein in *C. quinoa*

For GRAS proteins to function properly, they usually need to form complexes with other GRAS proteins or with other proteins. Previous reports have investigated that multiple GRAS factors can interact directly with DELLA, which clarifies DELLA’s diverse function in plants (Hirsch and Oldroyd, 2009; Locascio et al., 2013). In this family, interactions between two GRAS proteins, including NSP1, NSP2, SHR, and SCR, are also frequent (Cui et al., 2007; Hirsch and Oldroyd, 2009; To et al., 2020). For this reason, according to the orthologues’ link with *Arabidopsis thaliana*, we constructed the network of interactions between the quinoa protein (**Supplementary Table S6**).

**Figure 5:**
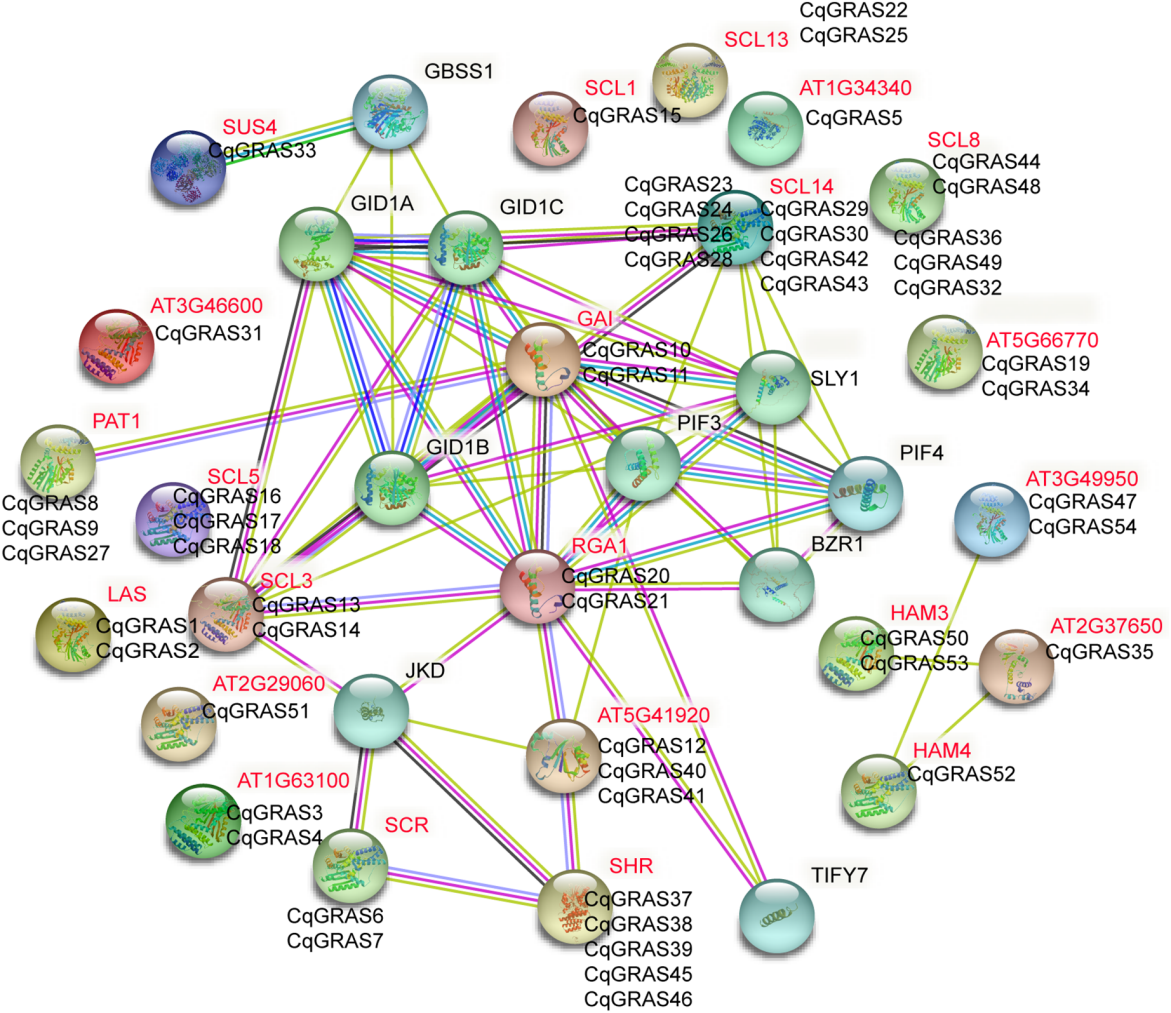
Quinoa’s CqGRAS protein functional interaction network based on *Arabidopsis* orthologues.

We identified 24 genes in *Arabidopsis thaliana*, 87.5% of which belong to the orthologues of the GRAS family of CqGRAS proteins (**Figure 5**), whereas no protein-protein interaction networks of AT1G34340, SUS4, SCL8, and AT3G46600 proteins were found in *Arabidopsis thaliana*. Based on the orthologous gene prediction of quinoa GRAS proteins in *Arabidopsis thaliana*, quinoa DELLA proteins (CqGRAS10, 21, 11, and 20) are gibberellin receptor proteins, and other CqGRAS proteins interact with transcription factors, including the WOX transcription factor family, which may mediate CqGRAS interaction. Our study further reveals the fact that the SHR-SCR-SCL13 module is the interaction center and connects with other modules by the interaction of SCL3-DELLA proteins. Our related network predictions provide clues for the study of the CqGRAS complex in quinoa, and these orthology-based predictions are helpful to study the action mechanism of the quinoa CqGRAS protein.

### 3.7 Expression analysis of *CqGRAS* genes in different tissues

**Figure 6:**
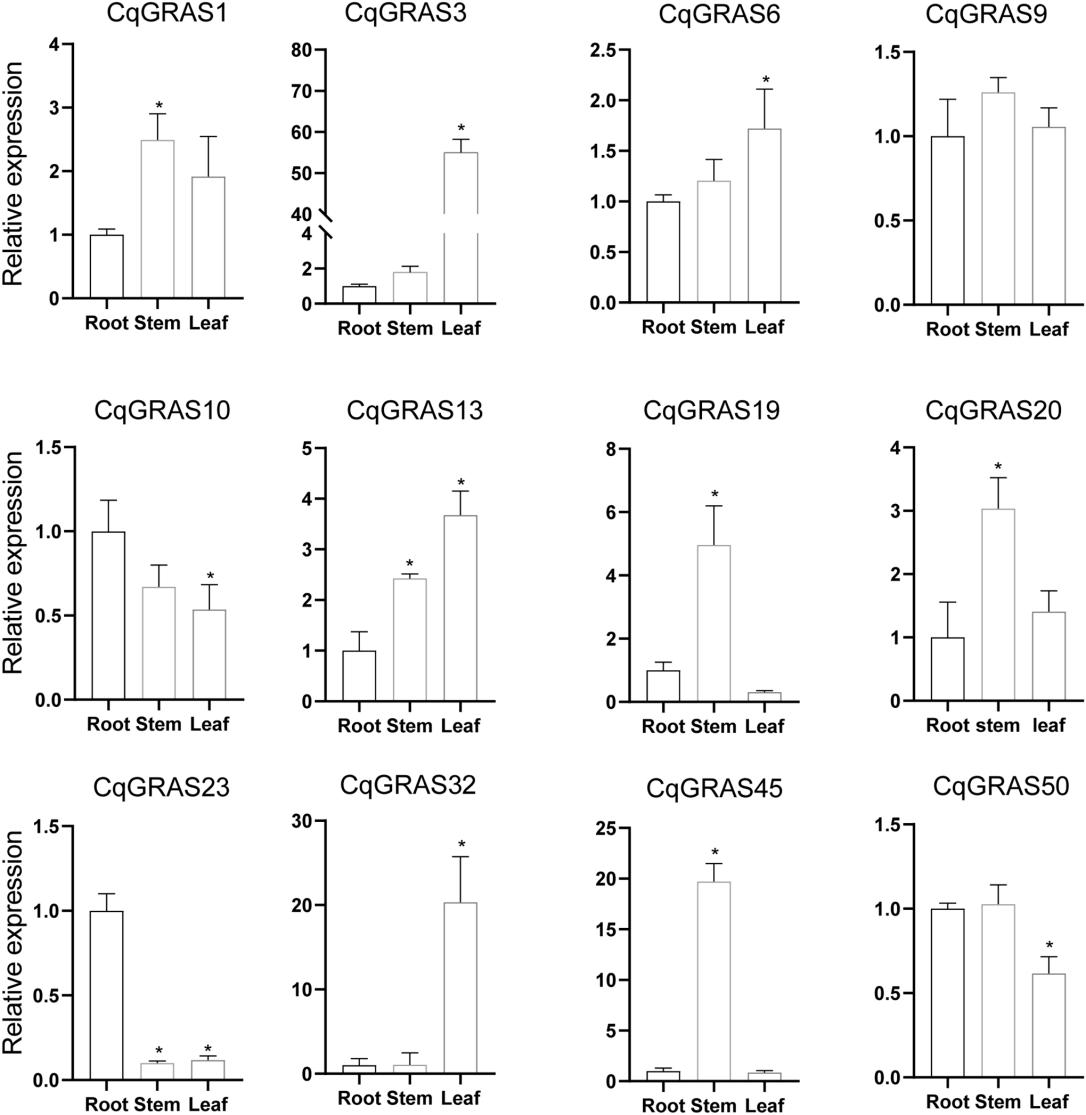
12 selected *CqGRAS* genes’ tissue-specific expression patterns using qRT-PCR. The X-axis depicts various tissues, such as the root, stem, and young leaf, while the Y-axis indicates the relative expression of *CqGRAS* genes. The expression level is relative to the root sample. Standard deviations for three replicates are represented by standard errors (bar), while *EF1α* served as an internal control. A significant difference (*P *< 0.05) between the expression levels of the root and other tissues is shown by the asterisk.

More and more data suggest that the *GRAS* genes play a fundamental role in plant growth and development (Khan et al., 2022). In this study, we screened 12 genes with potential research value (*CqGRAS1*, *CqGRAS3*, *CqGRAS6*, *CqGRAS9*, *CqGRAS10*, *CqGRAS13*, *CqGRAS19*, *CqGRAS20*, *CqGRAS23*, *CqGRAS32*, *CqGRAS45*, and *CqGRAS50*) for tissue-specific expression analysis in order to gain a better understanding of the function of the *CqGRAS* genes in quinoa (**Figure 6**). These genes have high sequence homology to other *GRAS* genes with known functions, especially in key conserved domains. Therefore, we speculated that they may play an important role in key processes of plant growth and development. Considering the limitations of experimental resources and time, we preferred these 12 genes for in-depth analysis. Among them, *CqGRAS1*, *9*, *19*, *20* and *45* genes are highly expressed in stems, and these genes may play an important role in stem development and growth. *CqGRAS3*, *6*, *13* and *32* genes are highly expressed in leaves, and these genes may be involved in leaf growth and photosynthesis-related processes. *CqGRAS10* and *23* genes are highly expressed in roots, which may be related to root development and adaptability to the soil environment. In addition, we observed that certain genes were not expressed in specific tissues: *CqGRAS23* was not expressed in stems, and *CqGRAS3* was not expressed in either stems or roots. The complex expression pattern of the *CqGRAS* genes in different tissues further confirmed that the *CqGRAS* genes play diverse functions in the growth and development of quinoa.

### 3.8 Responses of *CqGRAS* genes to different abiotic stress treatments

**Figure 7:**
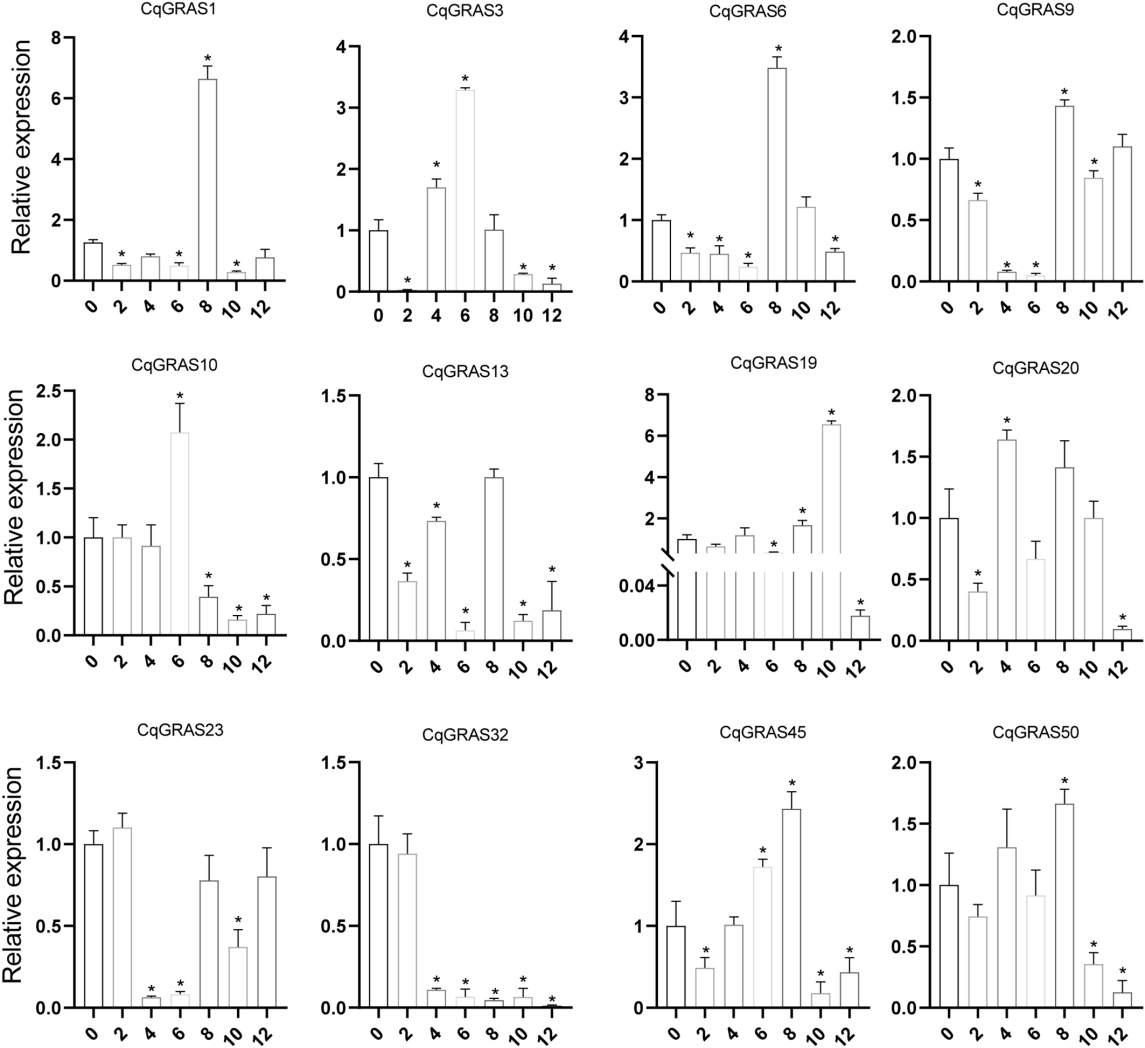
12 selected *CqGRAS* genes’ relative expression levels under abiotic stresses using qRT-PCR. *Chenopodium quinoa*’s gene expression patterns were analyzed under cold stress at seven different time points: 0, 2, 4, 6, 8, 10, and 12 hours. The control expression level at 0 h was normalized to 1. With three biological replicates, the standard error is displayed by the error bars. A significant difference (*P *< 0.05) in expression levels between the control and other time periods is shown by the asterisk.

*Chenopodium quinoa* has excellent resistance to low temperature. In order to assess the role of *CqGRAS* genes under low-temperature stress, we collected quinoa leaves under different time treatments and analyzed the expression of these 12 genes (**Figure 7**). The expression of six *CqGRAS* genes (*CqGRAS1*, *6*, *10*, *19*, *45*, and *50*) first increased but then decreased. The expression of four of those genes (*CqGRAS1*, *6*, *45*, and *50*) rapidly peaked at 8 h. The expression of the *CqGRAS10* gene peaked at 6 h, while the expression of the *CqGRAS19* gene peaked at 10 h. The expression of two *CqGRAS* genes (*CqGRAS9* and *23*) first decreased but then increased, reaching the lowest point at 6 h (*CqGRAS9*) and 4 h (*CqGRAS23*). The expression of one *CqGRAS* gene (*CqGRAS32*) gradually decreased. Last, *CqGRAS* genes showed different changes under low-temperature treatment, among which *CqGRAS1*, *19*, and *32* were the most sensitive to low temperature.

**Figure 8:**
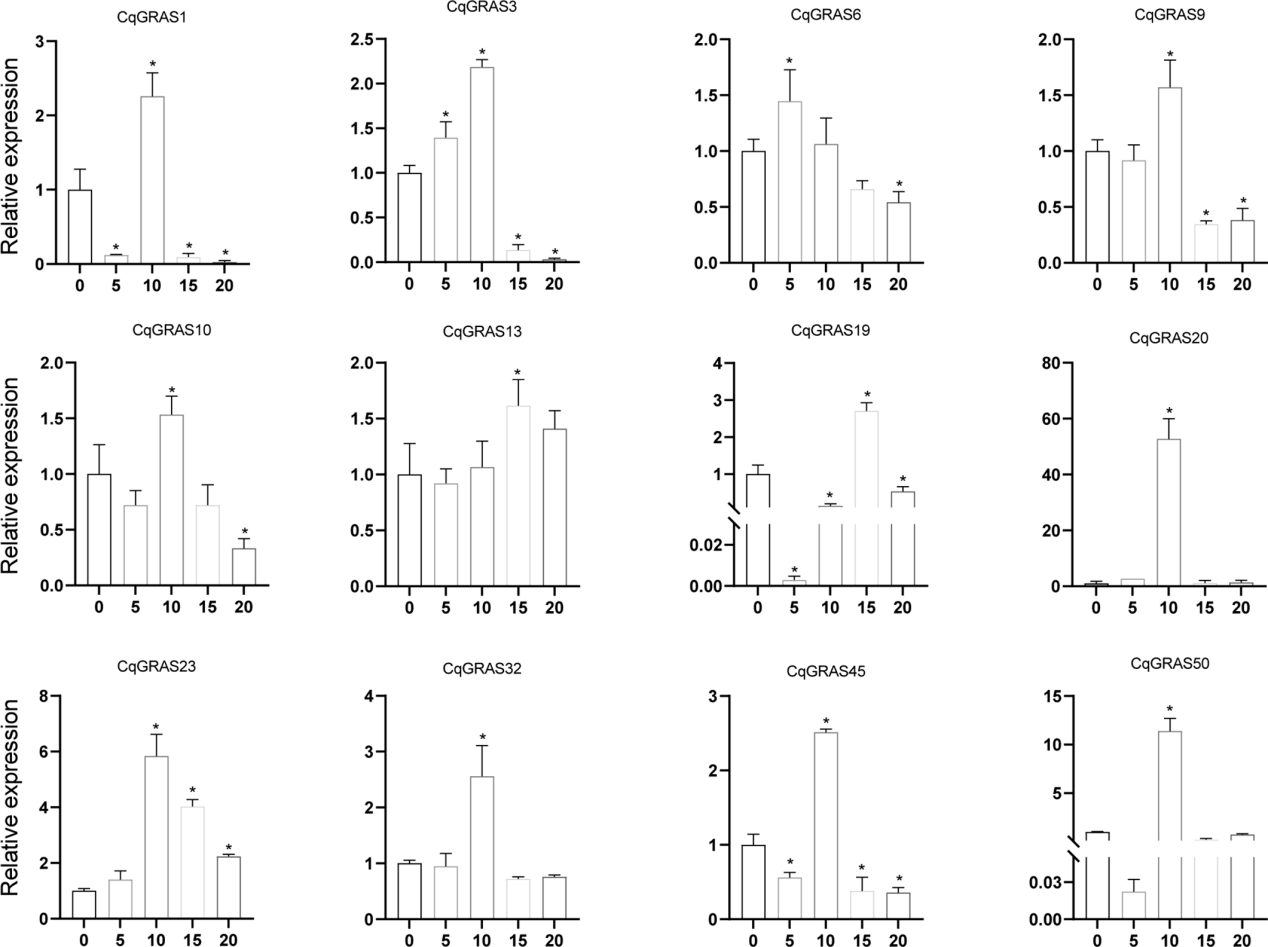
12 selected *CqGRAS* genes’ relative expression levels under abiotic stresses using qRT-PCR. At six hours, *Chenopodium quinoa*’s gene expression patterns were analyzed under stress conditions with varying H_2_O_2_ concentrations (0, 5%, 10%, 15%, and 20%). The control expression level was set to 1 at a hydrogen peroxide concentration of 0. With three biological replicates, the standard error is displayed by the error bars. A significant difference (*P *< 0.05) in expression levels between the control and other concentrations is shown by the asterisk.

Under the treatment of different concentrations of H_2_O_2_ (**Figure 8**), the expression of five *CqGRAS* genes (*CqGRAS1*, *10*, *19*, *45*, and *50*) first decreased, then increased, and finally decreased again; among them, the expression of four of those genes (*CqGRAS1*, *10*, *45*, and *50*) peaked at 10%, and the *CqGRAS19* gene peaked at 15%. The expression of seven *CqGRAS* genes (*CqGRAS3*, *6*, *9*, *13*, *20*, *23*, and *32*) first increased but then decreased; among them, the expression of five of those genes (*CqGRAS3*, *9*, *20*, *23*, and *32*) peaked at 10%, *CqGRAS6* reached the peak at 5%, and *CqGRAS13* peaked at 15%. The expression of *CqGRAS20* and *50* was highly induced under H_2_O_2_ treatment only at 10% concentration and maintained a low expression state at other concentrations.

**Figure 9:**
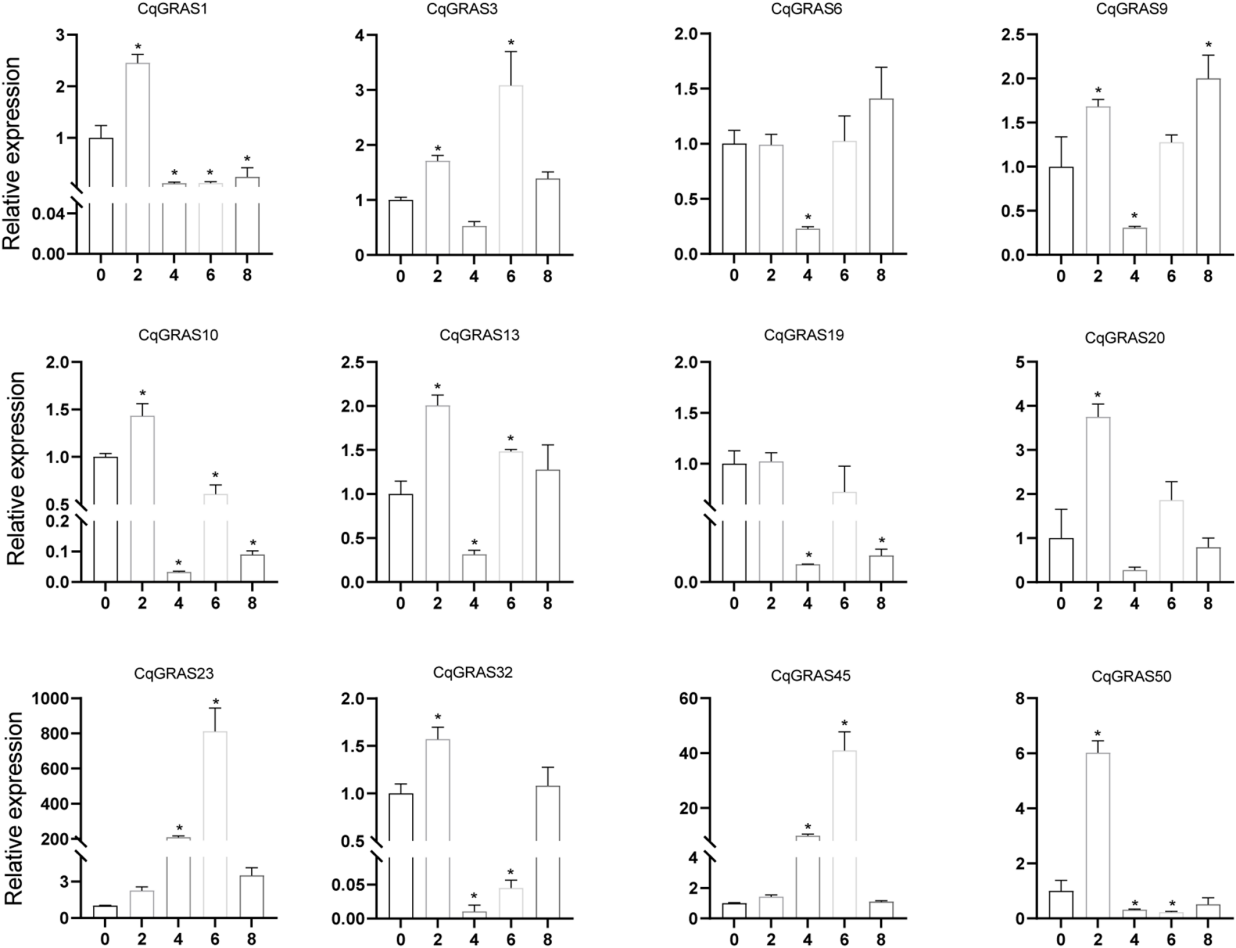
12 selected *CqGRAS* genes’ relative expression levels under abiotic stresses using qRT-PCR. At six hours, *Chenopodium quinoa*’s gene expression patterns were analyzed under stress conditions with varying Na_2_CO_3_ concentrations (0, 2, 4, 6, 8 mM). The control expression level was set to 1 at a sodium carbonate concentration of 0. With three biological replicates, the standard error is displayed by the error bars. A significant difference (*P* < 0.05) in expression levels between the control and other concentrations is shown by the asterisk.

*Chenopodium quinoa* often grows on acid-base imbalance soil, so we use Na_2_CO_3_ to treat quinoa to simulate acid-base imbalance soil (**Figure 9**). The expression of four *CqGRAS* genes (*CqGRAS1*, *23*, *45*, and *50*) first increased but then decreased; the expression of two of those genes (*CqGRAS1* and *50*) rapidly peaked at 2 mM, and the expression of the *CqGRAS23* and *45* genes peaked at 6 mM. The expression of the *CqGRAS6* gene first decreased but then increased, reaching the lowest point at 4 mM. The expression of two *CqGRAS* genes (*CqGRAS9* and *32*) first increased, then decreased, and finally increased again, reaching the lowest point at 4 mM. Interestingly, the expression of *CqGRAS23* and *45* was highly induced under Na_2_CO_3_ treatment.

## 4 Discussion

As a transcription factor, the *GRAS* gene is essential for plant growth, development, and stress response. Because of the excellent characteristics and stress resistance of *Chenopodium quinoa*, it is necessary to comprehend the role of the GRAS family. For this reason, we have examined information related to the GRAS family in order to investigate its evolution and speculate on the possible roles of unknown genes.

For this research, 54 quinoa GRAS proteins were identified, which was lower than that of wheat (Mishra et al., 2023), sorghum (Fan et al., 2021b), foxtail millet (Fan et al., 2021a), and rye (Fan et al., 2024), but higher than that of garlic (Zhang et al., 2022), oat (Pan et al., 2023), Chinese chestnut (Yu et al., 2022), and sugar beet (Hao et al., 2024). This change in the number of *GRAS* genes could be caused by genome size or gene duplication events (Grimplet et al., 2016). 16 pairs of segmental repeated *CqGRAS* genes and two sets of tandem repeat *CqGRAS* genes were found in this investigation. Quinoa *GRAS* amplification appears to be more influenced by segmental duplication than by tandem duplication. *CqGRAS* genes were located on all chromosomes and distributed unevenly, with the largest number of members on Chr07 and Chr18. In line with findings from other species, like oats and alfalfa, most other *GRAS* genes either contain one intron or no introns at all (80%), except for *CqGRAS5* and *33*, which is consistent with the lesser intron structure of cassava, tomato, and grape in previous studies (Huang et al., 2015; Grimplet et al., 2016; Shan et al., 2020). The *GRAS* gene family contains a large percentage of intronless genes, suggesting that GRAS members have a tight evolutionary relationship. Although intronless genes are typical in prokaryotic genomes, a study suggests that the GRAS family may have originated from bacterial infections between algae and moss (Neves et al., 2023). In the early stages of amplification, there are always a large number of introns that are slowly lost over time. Therefore, the more advanced the species, the fewer introns in the genome, which could account for why the *GRAS* gene has so many intronless formations.

According to the homology and classification of GRAS sequences in *Arabidopsis thaliana*, 54 *CqGRAS* genes were divided into 10 subfamilies: HAM, SHR, SCL3, LAS, SCR, DLT, SCL4/7, DELLA, PAT1, and LISCL (**Figure 1**). Although there are slight differences among different plant species, this family is consistent with the previous phylogenetic clustering, suggesting a rich diversity of *GRAS* genes in angiosperms (Liu and Widmer, 2014). The differences in conserved regions in 10 subfamilies indicate that each subfamily has different functions. In the process of evolution, functional separation can be caused by certain mutations of non-conservative amino acids. The CqGRAS family’s classification was further supported by the investigation of conserved motifs found in quinoa proteins. The protein motif arrangement and number of CqGRAS subfamilies are different, but the similarity among subfamilies is high. The motifs in the conserved domain of GRAS could serve essential roles. There are variations among CqGRAS members despite the fact that all of these proteins’ conserved motifs are similar; for example, motif 8 is common to all members, while HAM subfamily members do not have motif 3 or motif 6. These changes could be attributed to amino acid variations in non-conservative CqGRAS member regions, indicating that CqGRAS proteins have different functions in their microenvironments.

Earlier investigation indicates that *microRNA171* regulates certain GRAS members (Guo et al., 2019). Therefore, we predict the sequence of *microRNA171* binding sites based on the previously reported GRAS family members. The results show that there are 4 genes in the PAT1 subfamily that complement *Cq-microRNA171* (**Supplementary Figure S1**). There are also complementary sequences in other subfamilies, but there are differences in complementarity. This suggests that *microRNA171* may play a role in the regulation of specific *GRAS* genes in quinoa, possibly affecting their expression and function in different developmental processes and stress responses.

As observed in other species like sweet potato (Chen et al., 2019) and cassava (Shan et al., 2020), the GRAS transcription factor’s expression pattern varied in *Chenopodium quinoa* depending on the tissue. The gene expression data of 12 genes showed that the LISCL subfamily had low expression in stems and that the expression patterns of other subfamilies differed, as previously proved in alfalfa, *Brassica napus*, and pepper (Liu et al., 2018; Guo et al., 2019). DELLA protein is the main signal hub for controlling the growth and development of plants (Xue et al., 2022). Moreover, two DELLA subfamily genes (*CqGRAS10* and *20*) exhibit strong expression in a variety of tissues. The AtSCL4/7 subfamily’s *CqGRAS19* gene is expressed at a higher level in stems than in other tissues, indicating that the *CqGRAS* genes are important for stem development. The PAT1 subfamily’s *CqGRAS9* genes have high expression in leaves and stems, which may be related to AtSCL13, AtSCL21, and AtPAT1, participating in the signaling of phytochrome (Lu et al., 2024). Additionally, stems exhibit significant expression of *CqGRAS* genes from different subfamilies, which are possibly involved in a variety of developmental processes through photosensitive signal regulation systems (Khan et al., 2022). In short, these findings suggest that the function of the *CqGRAS* genes varies depending on the tissue.

*Chenopodium quinoa* has a strong ability to adapt to the growth environment of alpine and high altitudes (Yadav et al., 2023; Younis et al., 2023). The LTR cis-acting element is crucial for the cold stress response in plants, as it is an essential regulatory element for cold stress. Deletion of the element results in a complete loss of promoter activity, highlighting its crucial role in regulating gene expression under cold stress. About 45% of the members of the *CqGRAS* gene family have LTR elements, suggesting that the GRAS family is essential for quinoa’s reaction to cold stress. The expression analysis in this study revealed that the expression of major *GRAS* genes in quinoa was influenced by cold stress. Notably, two members of the DELLA subfamily in quinoa, *CqGRAS10* and *20*, exhibited elevated gene expression under low-temperature stress. This upregulation was associated with the presence of their own LTR promoter elements. Additionally, the expression levels of other subfamily genes containing LTR promoter elements changed significantly under low-temperature induction. This may indicate that multiple *GRAS* genes play different regulatory roles in quinoa against low-temperature stress. In addition to the LTR element, other cis-acting elements, such as ABA-responsive element (ABRE) and cold-responsive element (CRT/DRE), have also been shown to play a key role in regulating gene expression under abiotic stress. For example, ABRE elements play an important role in drought stress responses by binding to ABF transcription factors, regulating the expression of ABA-responsive genes (Feng et al., 2019; Zhu et al., 2025). Similarly, CRT/DRE elements are crucial in cold stress responses, and CBF transcription factors can bind to these elements to activate the expression of cold response genes (Tang et al., 2020; Song et al., 2021). These findings suggest that multiple cis-acting elements and their corresponding transcription factors collaborate to finely regulate the expression of stress-responsive genes in plants.

Quinoa often grows in acid-base imbalance and barren soil, where it is subjected to various stresses, such as oxidation and acid-base stress (Yadav et al., 2023). Quantitative RT-PCR analysis showed that quinoa *CqGRAS* genes responded differently to abiotic stresses. The *CqGRAS45* gene, a member of the AtSHR subfamily, was not only highly expressed in response to a range of abiotic stresses, particularly under conditions of acid-base imbalance, but also exhibited high expression in stems. This suggests that the AtSHR subfamily in quinoa may be involved in abiotic stress responses, rather than solely fulfilling its known role in stem development (Helariutta et al., 2000; Li et al., 2020). DELLA protein is crucial for controlling plant stress resistance (Zhao et al., 2021; Xue et al., 2022). *CqGRAS20*, a member of DELLA, is upregulated under acid-base imbalance and oxidation. According to expression studies, the majority of *GRAS* genes express level changes, indicating that *CqGRAS* genes are crucial for abiotic stress response. Overall, the quinoa *GRAS* gene’s potential regulatory role in the development and reaction to abiotic stress varies.

## 5 Conclusions

In this research, 54 *CqGRAS* genes found in the *Chenopodium quinoa* genome were divided into 10 subfamilies. The evolution of the *CqGRAS* gene family was driven by duplication and collinearity among members, and the distribution of these genes on 19 quinoa chromosomes was uneven. Detailed studies of gene structure, conserved motifs, and cis-acting elements provide insights into the potential regulatory mechanisms of these genes. Through expression analysis, it was found that certain *CqGRAS* genes (*CqGRAS20* and *23*) were significantly upregulated under abiotic stress. In addition, tissue-specific expression patterns were observed in which certain *CqGRAS* genes were highly expressed in specific tissues, such as stems or leaves. These results indicated that specific *CqGRAS* genes play a key role in coping with different abiotic stresses and may participate in various developmental processes of quinoa. Overall, our research provides a foundation for understanding the molecular mechanism behind the function of the *CqGRAS* gene family in stress response and development. The identified candidate genes with potential enhanced stress tolerance provide valuable resources for future genetic improvement work aimed at improving quinoa’s ability to adapt to adverse environmental conditions. Although valuable insights have been provided by bioinformatics analysis and expression analysis, direct validation of specific gene functions is lacking. To further support our findings, future research can validate the function of candidate genes through gene overexpression and gene knockout experiments, and conduct additional experimental validations, such as subcellular localization experiments. Gene overexpression and knockout experiments can be used to observe the phenotypic changes of quinoa plants under different environmental conditions, thereby directly assessing their role in stress responses and development. Subcellular localization experiments would help elucidate the cellular compartments where these *CqGRAS* proteins are active, providing insights into their potential roles in different cellular processes. These experimental methods will provide more specific evidence for the biological functions of the *CqGRAS* gene family and deepen our understanding of their role in quinoa’s adaptation to adverse environments.

## Data Availability

The original contributions presented in the study are included in the article/**Supplementary Material**. Further inquiries can be directed to the corresponding author.
