# Supplementary material for: Identification of two new GRAS transcription factors and expression analysis of these genes in Chenopodium quinoa
Source: Front Plant Sci. 2025 Jul 18;16:1579338. doi: 10.3389/fpls.2025.1579338 (PMC12313677; doi:10.3389/fpls.2025.1579338)
Supplement: Supplementary file 1 [file SupplementaryFile1.docx]

Supplementary Material

# Supplementary Figures
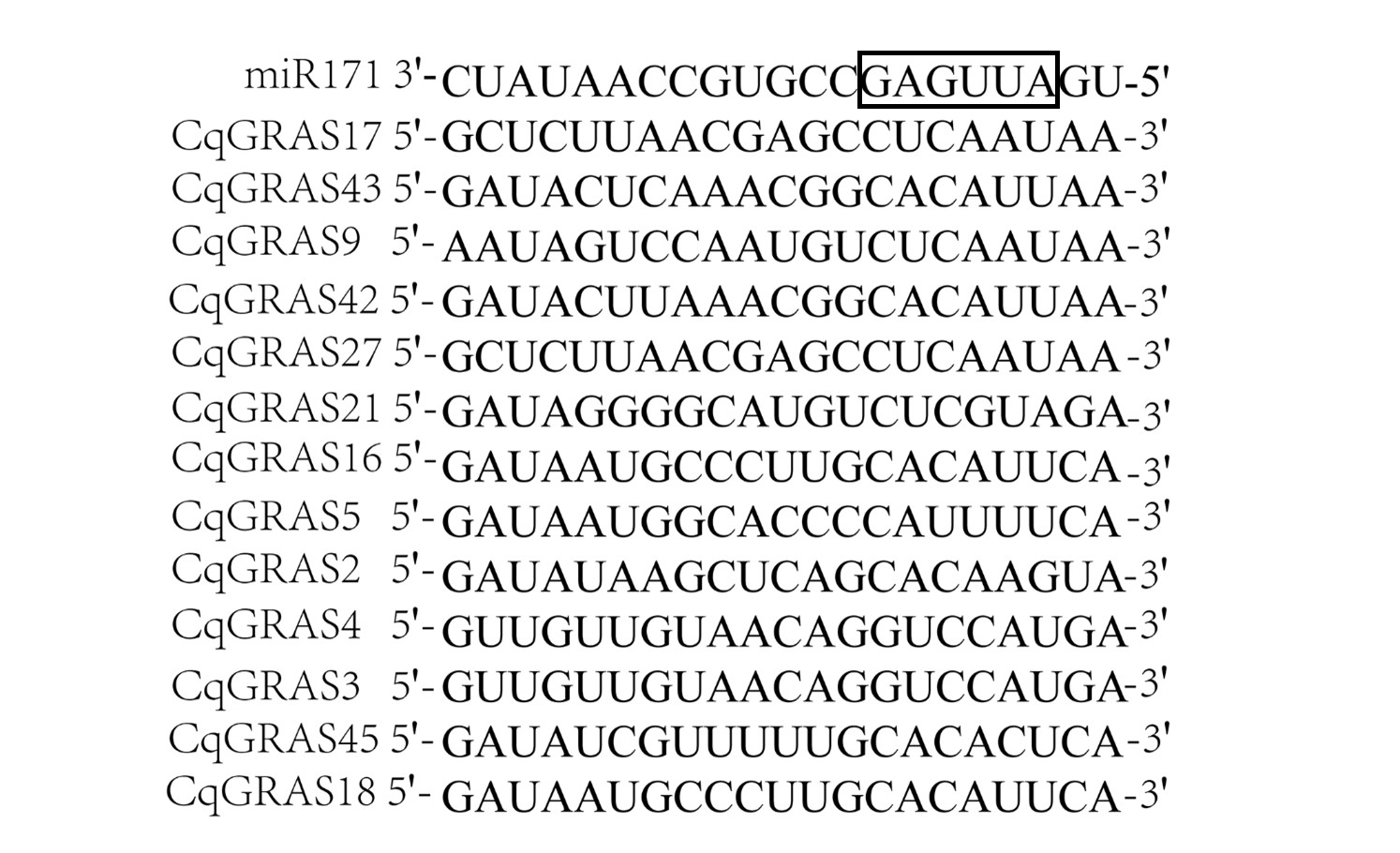


**Supplementary Figure 1.** Potential *GRAS* members targeted by *miRNA171* in *C. quinoa.* The region targeted by *miRNA171* is marked with a box in the figure.
